# Support for a tax increase to provide unrestricted access to an Alzheimer's disease medication: a survey of the general public in Canada

**DOI:** 10.1186/1472-6963-9-246

**Published:** 2009-12-29

**Authors:** Mark Oremus, Jean-Eric Tarride, Natasha Clayton, Parminder Raina

**Affiliations:** 1 McMaster Evidence-based Practice Center, McMaster University, DTC Building 3rd Floor, 1280 Main Street West, Hamilton, ON, L8S 4L8, Canada; 2 Department of Clinical Epidemiology and Biostatistics, McMaster University, 1280 Main Street West, Hamilton, ON, L8S 4L8, Canada; 3 Programs for Assessment of Technology in Health (PATH) Research Institute, St Joseph's Healthcare Hamilton, 25 Main Street West, Suite 2000, Hamilton, ON, L8P 1H1, Canada

## Abstract

**Background:**

Public drug insurance plans provide limited reimbursement for Alzheimer's disease (AD) medications in many jurisdictions, including Canada and the United Kingdom. This study was conducted to assess Canadians' level of support for an increase in annual personal income taxes to fund a public program of unrestricted access to AD medications.

**Methods:**

A telephone survey was administered to a national sample of 500 adult Canadians. The survey contained four scenarios describing a hypothetical, new AD medication. Descriptions varied across scenarios: the medication was alternatively described as being capable of treating the symptoms of cognitive decline or of halting the progression of cognitive decline, with either no probability of adverse effects or a 30% probability of primarily gastrointestinal adverse effects. After each scenario, participants were asked whether they would support a tax increase to provide unrestricted access to the drug. Participants who responded affirmatively were asked whether they would pay an additional $75, $150, or $225 per annum in taxes. Multivariable logistic regression analysis was conducted to examine the determinants of support for a tax increase.

**Results:**

Eighty percent of participants supported a tax increase for at least one scenario. Support was highest (67%) for the most favourable scenario (halt progression - no adverse effects) and lowest (49%) for the least favourable scenario (symptom treatment - 30% chance of adverse effects). The odds of supporting a tax increase under at least one scenario were approximately 55% less for participants who attached higher ratings to their health state under the assumption that they had moderate AD and almost five times greater if participants thought family members or friends would somewhat or strongly approve of their decision to support a tax increase. A majority of participants would pay an additional $150 per annum in taxes, regardless of scenario. Less than 50% would pay $225.

**Conclusions:**

Four out of five persons in a sample of adult Canadians reported they would support a tax increase to fund unrestricted access to a hypothetical, new AD medication. These results signal a willingness to pay for at least some relaxation of reimbursement restrictions on AD medications.

## Background

Alzheimer's disease (AD) is a neurodegenerative disorder that is characterized by progressive decline in cognitive and functional abilities. Early symptoms include loss of short-term memory, immediate event recall, and attention. Over time, patients lose the ability to perform activities of daily living [1]. The negative health impact of AD has been increasing relative to other chronic diseases in recent years. Between 2000 and 2006, deaths attributable to AD in the United States alone increased by 47%, while deaths attributable to heart disease, stroke, and prostate cancer decreased during the same time period [2]. Medications are the primary means of treating AD [3]. Existing medications have been shown to symptomatically treat cognitive decline for periods of up to one year, but they do not modify the course of disease [4]. Four medications are currently used to treat AD: donepezil, rivastigmine, and galantamine are part of a class of drugs called cholinesterase inhibitors (ChEIs); memantine is an N-methyl-D-aspartate receptor antagonist.

The modest efficacy of the four approved AD medications has led the administrators of public drug plans to develop evidence-based guidelines that generally permit patients to be reimbursed for the cost of these medications under certain conditions. For example, the Ontario Drug Benefit Program will reimburse donepezil for an initial three-month period if patients have a Mini-Mental State Examination (MMSE) [5] score of 10 to 26, which indicates mild or moderate AD. Continuation of reimbursement requires the MMSE to be re-administered at three-month intervals, with patients required to maintain a score between 10 and 26. Reimbursement may continue for a maximum period of one year [6]. Evidence suggests donepezil is not efficacious in persons with severe AD, so MMSE scores must be indicative of mild or moderate AD for reimbursement to continue.

Estimates from the Canadian Study of Health and Aging suggest that the prevalence of AD in the Canadian population will increase five-fold by 2031. Comparatively, the total Canadian population is expected to increase by a factor of only 1.4 during this same period [7]. Given the burden of AD on patients, caregivers, families [8], and society, reimbursement is an important issue for the public and policy makers to consider. Reimbursement is also important because AD is a chronic condition and the cost of drug treatment beyond a limited reimbursement period must be covered 'out-of-pocket'. Reimbursement will take on further importance as the first generation of disease modifying AD medications comes on the market in the next few years. However, it is currently unknown whether the general public would support funding unrestricted access to AD medications. This is especially important in countries like Canada, where the general public essentially funds the public healthcare system and forms the pool of future potential patients. To gauge the general public's view on reimbursement in light of these issues, and to guide policy makers in decision making, a national survey of adult Canadians was undertaken to assess the level of support for an increase in annual personal income taxes to fund a public program of unrestricted access to AD medications. To enrich the understanding of support for a tax increase, participants who said they would support the increase were asked about paying specific dollar amounts in tax.

## Methods

### Sample

The sample consisted of 500 Canadians, aged 18 years or over, who were chosen randomly from all 10 provinces. The sample was selected using ASDE Survey Sampler (ASDE Survey Sampler Inc., Gatineau, QC, Canada), which utilizes random digit dialling methodology to choose a sample of telephone numbers of potential participants.

Selection bias due to income could result if different proportions of high and low income groups participate in the survey and the likelihood of supporting a tax increase differs by group. To eliminate this potential selection bias, the sample was stratified into five income categories, each of which contained 100 participants. The five categories were: less than $20,000; $20,000 to less than $40,000; $40,000 to less than $60,000; $60,000 to less than $80,000; and $80,000 or more (all values in Canadian dollars).

### Survey

The data collection instrument was a two-part questionnaire. The first part contained questions on sample characteristics, e.g., age, sex, income, level of education, employment status. The first part also included the AD Knowledge Test (ADKT) [9] and the EQ-5D [10]. The ADKT has five true or false questions. Zero points are awarded for each 'true' response and one point is awarded for each 'false' response. Points are totalled and higher scores indicate a better knowledge of AD. The EQ-5D is a standardized instrument for measuring general health status, with questions on mobility, self-care, usual daily activities (e.g., work), pain and discomfort, and anxiety and depression. EQ-5D question responses were converted into an overall health utility index using United States population-based preference weights [11]. This index ranges from -0.11 (a health state worse than death) to 1.0 (perfect health). An index value of 0.0 is equivalent to death. The EQ-5D was administered twice in the questionnaire. For the first administration, participants were asked to rate their own health state today. For the second administration, participants were given a description of what may happen to persons with moderate AD (e.g., memory loss, increased dependence on caregivers) and they were asked to re-answer the EQ-5D under the assumption that they had moderate AD. Other questions in the survey included asking participants if they had any family members or close friends with AD, as well as whether family members or friends would approve of their decision to support a tax increase.

The second part of the survey consisted of four 'efficacy' scenarios describing a hypothetical, new AD medication. In two scenarios, the medication was described as treating the symptoms of cognitive decline. This is akin to the efficacy of the ChEIs and memantine. In the other two scenarios, the medication was described as halting disease progression (disease modification). For each pair of 'efficacy' scenarios, the medication was assumed to have no adverse effects in one scenario and a 30% chance of adverse effects in the other scenario. The adverse effects were described as primarily gastrointestinal in nature (e.g., vomiting, nausea, diarrhea).

For every scenario, participants were asked whether they would support unrestricted access to the medication through an annual increase in personal income taxes. Participants who responded affirmatively were asked in a separate set of questions whether they would pay $75, $150, or $225 per annum in additional personal income taxes. The middle value of $150 was the equivalent Canadian cost of supplying one person with existing, approved AD medications for one month. Only participants who indicated they would support unrestricted access were asked about specific dollar amounts (bids) to avoid 'yeah saying' bias, where someone would accept any bid as a means of expressing support for the program in question [12]. In the event a participant did accept all three bids, an open-ended question was asked to elicit the maximum amount they would be willing to pay. For participants who supported a program of unrestricted access and rejected each of the three bids, an open-ended question was asked to obtain a value between $0 and $74.

Our process of presenting bids to participants was based on the contingent valuation literature's dissonance minimizing methodology (coupled with a bid range). Dissonance minimization involves the use of two sets of questions: a preliminary question without a bid value to ascertain whether participants support a program and a follow-up question to assess whether participants support a specific bid. Participants who answer the preliminary question affirmatively will move on to answer the bid question. The purpose of this tiered approach is to avoid yeah-saying bias. In another method, known as dichotomous choice, participants are presented with a single question containing a bid that they accept or reject. We avoided dichotomous choice to guard against yeah-saying bias.

In both dissonance minimization and dichotomous choice, each participant is often presented with a single bid. Thus, large sample sizes are required to obtain ranges of support across different bids. We reduced the sample size requirement by asking each participant to accept or reject a range of different bids.

### Survey design and administration

The content of the survey was developed by the authors in accordance with the results of a recent systematic review of AD studies in the contingent valuation literature [13]. The wording of the questions and response options was based on the principles of survey design [14]. The survey was pretested on 45 members of the general public. Interviewers administered the pretest over the telephone as though they were doing 'real' interviews. The purpose of the pretest was to note and correct challenges with question flow, skip patterns, and response options, as well as to elicit participant feedback on issues such as unclear question wording.

The final version of the survey was administered by trained interviewers to study participants via Computer-Assisted Telephone Interviewing (CATI) technology. Participants were randomized to the order of scenarios and to the order of bids to prevent ordering and starting point bias. A minimum of seven attempts was made per telephone number to complete the interview, after which the contact was classified as a non-respondent.

Since the survey was administered to French-speaking persons in the Province of Québec, the final English-language version of the survey was translated into French. The translation was verified by a bilingual co-author (JET). A copy of the English version of the survey is included as an appendix (see Additional file 1).

The study protocol and survey received ethics approval from the Hamilton Health Sciences/McMaster Faculty of Health Sciences Research Ethics Board (reference number: 08-179).

### Statistical analysis

Descriptive statistics were calculated for all variables. These statistics included frequencies for categorical variables and medians and 25^th ^to 75^th ^percentile ranges for continuous variables.

A logistic regression analysis was conducted to examine the determinants of support for a tax increase. The primary outcome variable was dichotomous: a participant supports a tax increase under at least one scenario or does not support a tax increase under any scenario. This variable was chosen to assess participants' overall openness to the idea of supporting a tax increase. Based on the aforementioned review of contingent valuation papers in AD [13], 11 possible explanatory variables were selected for use in regression modeling (Table 1). Continuous variables were assessed to see if they were linear in the logit using the procedure outlined by Hosmer and Lemeshow [15]. If not, then these variables were categorized at the quartile(s) where the direction of effect appeared to change.

**Table 1:** Sample characteristics

Characteristic	n	%
Sex		
Female	305	61
Male	195	39
Annual household income		
<$20,000	100	20
$20,000 to <$40,000	100	20
$40,000 to <$60,000	100	20
$60,000 to <$80,000	100	20
≥ $80,000	100	20
Region of residence		
Atlantic	36	7
Québec	155	31
Ontario	167	33
Prairies	83	17
British Columbia	59	12
Highest level of education completed		
High school or less	169	34
At least some technical or community college	143	29
At least some university	184	37
Missing	4	<1
Employment status		
Not working (unemployed, student, homemaker)	66	13
Retired	142	28
Employed (full- or part-time)	281	56
Missing	11	2
Participant knows a family member or close friend with Alzheimer's disease		
Yes	211	42
No	286	57
Missing	3	<1
Family members' or friends' level of approval of a participant's decision to support a tax increase*		
Strongly disapprove	39	8
Somewhat disapprove	78	16
Somewhat approve	260	52
Strongly approve	110	22
Missing	13	3
Alzheimer's disease knowledge score^†^	4**	3 to 4^††^
Age^‡^	51**	40 to 64^††^
EQ-5D Index (current health state)^¶^	0.83**	0.80 to 1.00^††^
EQ-5D Index (participants assume they have Alzheimer's disease)^§^	0.68**	0.47 to 0.77^††^

*This is the participant's belief in whether a family member or friend would approve of their decision to support a tax increase. It is not the participant's actual knowledge of whether family or friends would approve.

^†^Not linear in the logit: categorized for regression analyses as good (score = 4 to 5), fair (score = 2 to 3), poor (score = 0 to 1).

^‡^Not linear in the logit: categorized for regression analyses as <65 years, ≥ 65 years.

^¶^Not linear in the logit: categorized for regression analyses as <0.84, ≥ 0.84.

^§^Not linear in the logit: categorized for regression analyses as <0.78, ≥ 0.78.

**Median.

^††^25^th ^to 75^th ^percentile.

Univariable logistic regression analyses were conducted to examine the effect of each individual variable in Table 1. A full, multivariable logistic regression model was built using the following rules: age, sex, and income were forced into the model regardless of their levels of significance in the univariable regression analyses; the remaining variables were included in the full model if their p-values (Wald chi-square) were ≤ 0.20 in the univariable regression analyses. The full model was paired down into a reduced model. The following rules were used to determine the variables that would be retained in the reduced model: age, sex, and income were automatically retained; variables with a p-value < 0.05 in the univariable regression analyses; and variables with a p-value < 0.05 in the full model.

The full and reduced models were compared with one another using the likelihood ratio test and Akaike Information Criterion (AIC). Model fit was assessed using the Hosmer and Lemeshow Goodness-of-fit test. Variables were considered statistically significant at the 5% level (α = 0.05). SAS v9.1 (The SAS Institute, Cary, NC) was used to conduct all statistical analyses.

We used McNemar's test to compare whether the number of participants in each scenario who accepted bids other than $75 was statistically significantly different from the number who accepted the $75 bid.

A series of exploratory analyses were also conducted to investigate whether the determinants of support for a tax increase differed by individual scenario. For each of the four scenarios, full and reduced multivariable logistic regression models were built using the same steps as outlined above.

## Results

### Sample characteristics and descriptive statistics

A total of 13,195 unique telephone numbers were dialled to obtain 500 participants. Out of the total dialled, 8,718 (66%) did not generate participants for 'process' reasons (e.g., no answer, busy signal, not in service), 2,460 (19%) led to persons who refused participation or terminated an in-progress interview, 273 (2%) led to persons who were rejected because the 100-participant income quota for the stratum into which they fit had been reached, 92 (<1%) led to persons who refused to provide their income, and 1,112 (8%) resulted in scheduled call-backs that were unneeded after having already achieved the required sample size. The remaining 540 telephone numbers (4%) led to completed interviews. The 40 interviews above the required 500 were conducted to correct a regional sampling disparity, which produced no initial representation from Québec in the highest income stratum. After the 40 additional interviews, the highest income stratum contained 140 participants, out of which 100 were chosen via a simple random sample for inclusion in the analysis.

The median age of the 500 participants was 51 years (25^th ^to 75^th ^percentile range: 40 to 64) and 61% were female. Thirty-seven percent had at least some university education, 57% were employed full- or part-time, and 42% knew a family member or close friend with AD. Table 1 shows more characteristics of the sample.

**Figure 1:**
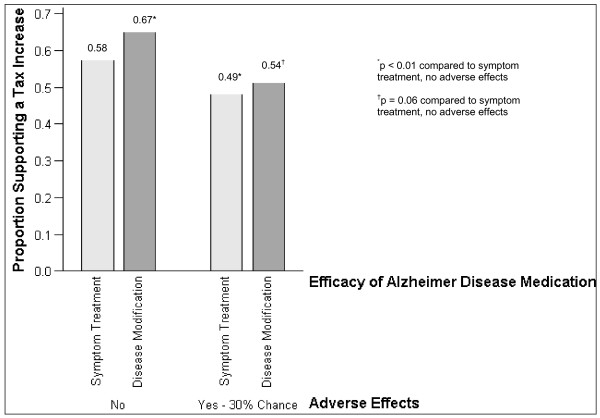
**Support for an increase in annual personal income tax to fund a program of unrestricted access to Alzheimer's disease medications -- specific scenarios**. *Proportion of participants in favour of support for an increase did not differ by order of randomization: *Symptom treatment, no adverse effects:  = 29.62, p = 0.16. Symptom treatment, 30% chance of adverse effects:  = 23.07, p = 0.42. Disease modification, no adverse effects:  = 22.15, p = 0.51. Disease modification, 30% chance of adverse effects:  = 32.67, p = 0.09.

Eighty percent of participants (n = 398) supported a tax increase for at least one scenario. Support for a tax increase at the level of specific scenarios ranged from 49% to 67% (Figure 1). Support was highest for the most favourable scenario (disease modifying medication without adverse effects) and lowest for the least favourable scenario (medication treats symptoms only and has a 30% chance of adverse effects). When stratified by adverse effects profile (no adverse effects versus 30% chance), support was highest for the disease modifying version of the medication. When stratified by type of efficacy, support was highest for the medication without adverse effects.

In univariable logistic regression analyses, five variables had p-values ≤ 0.20: AD knowledge score, EQ-5D index scores for participants' current health states and their assumed health states if they had AD, whether they knew a family member or close friend with AD, and whether family or friends would approve participants' decisions to support a tax increase. The full model included these five variables and age, sex, and income. The reduced model included all of the variables from the full model except AD knowledge score and the EQ-5D index score for participants' current health states.

In the full and reduced logistic regression models for supporting a tax increase under at least one scenario, the only two variables that were statistically significant at the 5% level were EQ-5D index score (participants assume they have moderate AD) and relatives' or friends' approval of participant support for a tax increase. Although both the full and reduced models were a good fit to the data, the full model was preferred over the reduced model based on the likelihood ratio test and AIC. The results of both models are summarized in Table 2.

**Table 2:** Multivariable logistic regression analysis -- participant support for a tax increase under at least one scenario

	Models	
Variables	Full OR (95% CI)	Reduced OR (95% CI)
Alzheimer's disease knowledge score		
Good	0.27 (0.03-2.46)	NIM
Fair	0.24 (0.03-2.15)	NIM
Poor	1.00	NIM
Age		
≥ 65 years	1.15 (0.56-2.33)	1.25 (0.63-2.49)
< 65 years	1.00	1.00
Sex		
Female	0.99 (0.57-1.71)	0.96 (0.56-1.64)
Male	1.00	1.00
EQ-5D Index (current health state)		
≥ 0.84	1.25 (0.73-2.14)	NIM
< 0.84	1.00	NIM
EQ-5D Index (participants assume they have Alzheimer's disease)		
≥ 0.78	0.47 (0.26-0.85)	0.43 (0.24-0.77)
< 0.78	1.00	1.00
Annual household income		
≥ $80,000	1.04 (0.41-2.62)	1.19 (0.49-2.88)
$60,000 to < $80,000	1.10 (0.44-2.72)	1.25 (0.52-2.99)
$40,000 to < $60,000	0.48 (0.21-1.09)	0.53 (0.24-1.16)
$20,000 to < $40,000	1.15 (0.36-2.09)	1.09 (0.48-2.50)
< $20,000	1.00	1.00
Family/friend has Alzheimer's disease*		
Yes	1.34 (0.77-2.30)	1.28 (0.75-2.19)
No	1.00	1.00
Family/friend approval^†^		
Strongly approve	13.49 (4.30-42.33)	14.72 (4.80-45.21)
Somewhat approve	4.57 (1.96-10.61)	4.82 (2.12-10.95)
Somewhat disapprove	1.50 (0.61-3.71)	1.66 (0.69-4.02)
Strongly disapprove	1.00	1.00
-*2 Log likelihood*	361.92	369.94
*Likelihood ratio test* *(reduced model versus full model)*	*G = 8.02; = 7.82* *p = 0.0456*	
*Akaike information criterion*	391.92	393.94
*Hosmer and Lemeshow Goodness-of- fit Test*	*p = 0.4431*	*p = 0.3039*

OR = odds ratio; CI = confidence interval; NIM = not in model; G = difference in -2 log likelihoods between models.

*Participant knows a family member or close friend with Alzheimer's disease.

^†^Family members' or friends' level of approval of a participant's decision to support a tax increase.

Based on the models in Table 2, the odds of supporting a tax increase under at least one scenario were approximately 55% less for participants who attached higher ratings to their health state (i.e., EQ-5D ≥ 0.78) under the assumption they had moderate AD, relative to participants who attached lower ratings (i.e., EQ-5D < 0.78). If participants thought family members or friends would somewhat or strongly approve of their decision to support a tax increase, then the odds of actually supporting a tax increase under at least one scenario would increase by approximately five times or more, relative to situations where family or friends were thought to strongly disapprove. The odds ratios and confidence intervals for these two variables were consistent across both the full and reduced models.

In the exploratory regression analyses pertaining to determinants of support for a tax increase under each individual scenario (Table 3), family members' or friends' approval of a participant's decision to support a tax increase was statistically significant at the 5% level in all full and reduced models. The results were consistent across the four scenarios and the odds ratios were similar to the models shown in Table 2. EQ-5D score under the assumption that participants had moderate AD was significant at the 5% level in the scenario involving a medication that treats symptoms and has no adverse effects. The direction of effect for EQ-5D score was negative (odds ratio [OR] = 0.57; 95% confidence interval [CI] = 0.34 to 0.93 [reduced model]). Participants aged 65 years or more were less likely than participants aged less than 65 years to support a tax increase when the medication treated symptoms and had a 30% chance of adverse effects (OR = 0.58; 95% CI = 0.32 to 0.99 [reduced model only]).

**Table 3:** Exploratory analyses: summary of regression models

Variable	S1		S2		S3		S4	
	FM	RM	FM	RM	FM	RM	FM	RM
Alzheimer's disease knowledge score							√	√
Age				√			√	√
Sex								
EQ-5D Index (current health state)								
EQ-5D Index (participants assume they have Alzheimer's disease)	√	√						
Annual household income*							√	√
Region of residence								
Highest level of education completed								
Employment status								
Participant knows a family member or close friend with Alzheimer's disease								
Family members' or friends' level of approval of a participant's decision to support a tax increase^†^	√	√	√	√	√	√	√	√

S1 = scenario 1 (symptom treatment, no adverse effects); S2 = scenario 2 (symptom treatment, 30% chance of adverse effects); S3 = scenario 3 (disease modification, no adverse effects); S4 = scenario 4 (disease modification, 30% chance of adverse effects); FM = full model; RM = reduced model; √ = variable is significant at the 5% level (p < 0.05) in the model.

*Only the '$60,000 to less than $80,000' category (versus 'less than $20,000') is significant at the 5% level.

^†^Only the 'somewhat approve' and 'strongly approve' categories (versus 'strongly disapprove') are significant at the 5% level.

For the scenario involving a disease modifying medication with a 30% chance of adverse effects, three variables explained support for a tax increase in addition to 'family member or friend approval' (all results from reduced model): good versus poor knowledge of AD (OR = 0.17; 95% CI = 0.03 to 0.90) and fair versus poor knowledge of AD (OR = 0.15; 95% CI = 0.03 to 0.78); age 65 years or more versus less than 65 years (OR = 0.45; 95% CI = 0.25 to 0.80); and income of $60,000 to less than $80,000 versus less than $20,000 (OR = 2.37; 95% CI: 1.17 to 4.81). Table 3 shows the variables that were significant at the 5% level in the scenario-specific models. Specific model details are available from the authors upon request.

**Figure 2:**
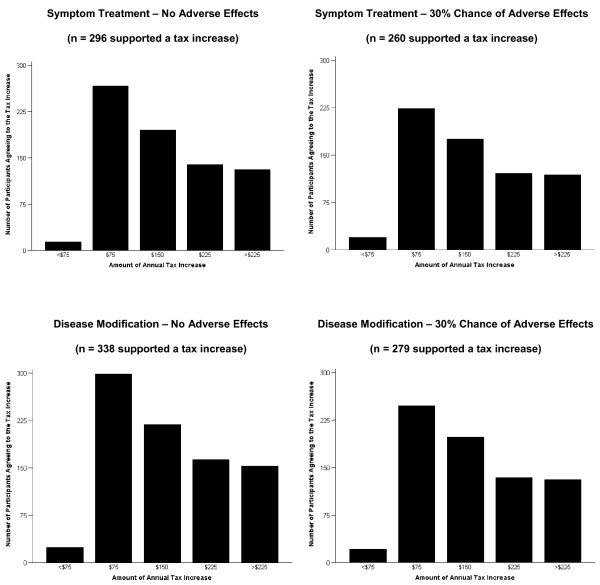
**Number of participants agreeing to specific annual income tax increases to fund a program of unrestricted access to an Alzheimer's disease medication**. Participants may have agreed to more than one of the values in each scenario. The number of participants in each scenario who agreed to values other than $75 was statistically significantly different (P < 0.05 [McNemar]) from the number who agreed to $75.

Among the participants who supported a tax increase under each scenario, at least 86% reported they would pay $75 extra per annum for a program of unrestricted access to AD medications. Approximately two-thirds would also pay $150, and just under half would pay $225 (Figure 2; p < 0.05 for all dollar-value comparisons against $75).

## Discussion

Eighty percent of participants supported a tax increase, under at least one of four scenarios, to fund unrestricted access to a hypothetical, new AD medication. Support was highest for the most favourable scenario, in which the medication was described as modifying the course of disease without entailing adverse effects.

A majority of participants would be willing to pay an additional $150 per annum in personal income taxes to fund unrestricted access to the medication. Just under half of the participants would also be willing to pay more than $225 per annum. For an average, single Canadian without children who earns $40,000 per annum and has an annual tax burden of 25% of income (i.e., $10,000 paid in taxes) [16], an extra $150 would translate into a 1.5% increase in tax burden.

This is the first study in Canada or elsewhere to assess whether the general public would pay more taxes to liberalize public reimbursement regimes for AD medications. In a recent systematic review [13], two of the authors (MO, JET) could only find one other study of taxation and publicly-subsidized programs in AD. This study, by Nocera et al. [17], involved 1,240 German-speaking persons in Switzerland who were aged 18 years or more and selected randomly from a telephone book. Three AD programs were assessed: two-day training for unpaid caregivers and free access to a nurse professional for four weeks annually; early screening; and intensified research in Swiss universities. Participants' support was assessed for various specific levels of taxation, e.g., would a participant support a tax increase of 'X' Swiss francs? Support was highest for the 'training-and-nurse' program (specific level of support not reported), followed by research (80% support) and screening (20% support). Nocera et al. did not report the percentage of participants who supported at least one program. The Swiss study, like the current study, shows that members of the general public do support increased taxation for AD programs, with the level of support varying depending on the perceived value of the program.

Subsequent to the review, a study by Negrín et al. [18] was published involving 598 participants selected randomly from the adult population of the Canary Islands. The participants were presented with a mix of three distinct programs (i.e., home care, access to day care centres, access to medium- and long-stay centres), combined with a mix of 'monthly contributions' (tax increases) to fund each program (i.e., €12, €24, €48, €72). The mix of programs and contributions was varied for each participant, who was asked to select the most preferred option from the mix. Home care was preferred by the majority of participants. Only 77 participants (13%) said they would not pay anything for these programs.

From a policy making perspective, the results suggest that Canadians would be likely to support (and pay taxes for) at least some relaxation of reimbursement restrictions on AD medications. The most support would flow when medications modify disease, especially in the absence of adverse effects. This finding is particularly relevant because the first disease modifying medications are due on the market within the next few years. Of course, if a disease modifying medication without adverse effects were ever to come on the market, then policy makers would likely approve it and may even offer unrestricted reimbursement, regardless of public opinion.

This study of the Canadian general public has numerous strengths that lend credence to its findings. First, the participants were recruited from a pan-Canadian sampling frame using a random sampling methodology, thus eliminating selection biases associated with region of residence or location of recruitment (e.g., recruiting visitors to doctors' offices instead of recruiting a broader, national sample). Second, data were collected with a standardized interview that was conducted by trained interviewers using CATI software to lessen potential information bias. Third, the order of scenarios, starting bids, and bid ranges were randomized to prevent ordering or starting point bias. Ordering bias can occur when participants' answers to a scenario are influenced by their answers to preceding scenarios, while starting point bias can occur when respondents express WTP values that are close to the initial bids. In an effort to reduce yeah saying and protest answers, respondents were first asked if they would support a tax increase. Fourth, the results for supporting a tax increase for specific scenarios made intuitive sense. Support was highest for the optimal drug scenario (disease modification, no adverse effects) and lowest for the least favourable scenario (symptomatic treatment, 30% chance of adverse effects). Support was also highest for the smallest tax increase ($75) and lower for larger tax increases (> $75) (Figure 2; p < 0.05 for all dollar-value comparisons versus $75).

In this study, the variable that was most consistently associated with support for a tax increase to fund unrestricted access to AD medications was participants' perceptions about whether family members or friends would somewhat or strongly approve of their decision to support a tax increase. Perhaps the favourable views of persons who are at the core of one's social network could exert a positive influence on support for a tax increase. Werner et al. [19] refer to this as 'subjective norm' and found similar results in a study of 220 caregivers of family members with AD. The caregivers in the Werner et al. study were found to be willing to pay more for an AD medication when they believed their close associates would approve of their paying for the medication.

Income was not associated with support for a tax increase. Three reasons could explain this finding. First, effects were undetectable because the sample was stratified into five equal-sized income categories to control for possible selection bias on income. Second, participants' said 'yes' to the tax support questions to 'do the right thing' or please the interviewer by giving a socially desirable response. Third, participants found the program affordable regardless of income.

Interestingly, 42% of the participants in the Canadian general public survey knew someone with AD. This may not be representative of the general Canadian population. Additionally, participants' median age was 51 years and 23% were 65 years or older, which indicates that the participants were an older subgroup of the Canadian population (2006 Census: median age = 39.5 years; percentage aged 65 years or more = 13.7% [20]). Participant characteristics on both variables could suggest that the study's findings overestimated the percentage of Canadians who would support a tax increase. For example, participants who know someone with AD will be aware of the devastation caused by the disease, thus enhancing their predisposition to support a tax-funded program of unrestricted access to AD medications. An older sample has an elevated risk for AD. These persons may recognize the challenges of being on a fixed income when their risk is highest, so they may be more likely to support a program of free access to medications.

The potential for overestimation of support for a tax increase is not actually born out by the data. In multivariable regression analyses, the crude association between knowing someone with AD and support for a tax increase was eliminated after adjustment for other possible explanatory variables. Age is inconsistently associated with support and it is significant at the 5% level in only three models that were built for the exploratory analyses. In these models, age was inversely associated with support. Perhaps people in upper middle age (who were saving for retirement) and seniors on fixed incomes were reticent to support a program that would reduce their personal financial resources. These persons might have made implicit risk-benefit calculations and concluded that the risk of actually getting AD was outweighed by the need to save money for present or future personal expenditures.

The data in this study are cross-sectional. Therefore, the direction of effect between some of the variables may be the reverse of what is hypothesized in the study (reverse causality). For example, it is possible that a participant's close family or friends might know that she or he supports a tax increase. Such knowledge could prompt the acquaintances to approve the participant's choice after it has been made. This is the opposite of the hypothesized direction of effect, which suggests the participant's perception of family or friends' approval influences the decision to support a tax increase.

The issue of reverse causality does not apply to all of the variables, simply due to their nature. For example, a participant's age and sex will not be affected by whether she or he supports a tax increase. Likewise, the number of relatives or friends with AD will probably be unaffected by one's support, or lack of support, for a tax increase. The same would be the case for health status on the EQ-5D, employment status, education, knowledge of AD, region of residence, or income.

The 4% overall interview completion rate (more than 13,000 telephone numbers dialled to generate 500 interviews) must be viewed in the context of the random digit dialling method. The ASDE Survey Sampler dialled random telephone numbers without guarantee that someone would answer the phone. Out of 13,195 dialled numbers, 66% were not answered, were busy, or were not in service and did not result in a contact. Since public opinion on tax support for AD medications is unlikely to be associated with answering a telephone, the potential for bias in this regard is likely to be minimal. For similar reasons, bias is also unlikely in the case of persons who were rejected because their income fell within a stratum for which the 100-participant quota was reached or because they were unneeded after the overall sample size requirement was met. Greater potential for bias might result from persons who refused participation or terminated an in-progress interview (n = 2,460), or who refused to provide their income (n = 92). Inclusion of these two groups in the denominator along with persons who agreed to participate (n = 540) creates a more realistic response proportion of 17%. Reasons for refusing to participate or for hanging-up are many and may well be unrelated to support for a tax increase (e.g., poor timing of the telephone call, person who answers the phone never as a rule participates in surveys, person perceives that the interview is taking too long and terminates the call). Although the participants were more likely to know someone with AD or be older than the average Canadian, these traits did not appear to bias the study results. Similarly, the decision to refuse to provide an income may not be associated with one's level of support for a tax increase. Indeed, the potential confounding effects of income were addressed by enrolling an equal number of participants in five income strata.

Certain segments of the population will be excluded from the sampling frame of a random digit dialling survey because they do not have a land line-based telephone. These persons include the homeless, people living in institutions, and people who use only cellular telephones. The possibility of bias in excluding these persons is likely to be minimal. The homeless or persons who are institutionalized due to mental or cognitive impairments would not be in a position to make decisions about supporting a tax increase, nor might they even pay taxes as a result of their circumstances. The exclusion of persons who use only cellular telephones would bias the results if they are more or less likely as a group, compared to people with land lines, to support a tax increase. Currently, there is no evidence to suggest whether the 'cellular only' group would be different from the 'land-line' group in this respect.

A potential issue in many surveys is social desirability bias, where participants provide what they believe are socially acceptable answers to questions or provide responses that they believe the interviewer wants to hear. While it is possible that this bias led to an overestimate of support for a tax increase, it should be noted that participants were identified through a telephone number, not by name, and they remained anonymous throughout the survey. Also, participants had no in-person contact with interviewers. The anonymous, impersonal nature of the interviews may have lessened participants' impetus to provide socially desirable responses because they would not have had to share unpopular views with peers. Additionally, non-verbal cues from the interviewer (e.g., nods of approval, frowns) would not be evident to participants over the telephone.

The models shown in Table 3 are dissimilar from one another. Some of the dissimilarity may be the result of the different sample sizes used to estimate the scenario-specific models. Sample sizes were based on the number of participants who said they would support a tax increase under each scenario. It is also possible that the determinants of support for a tax increase differ across scenarios. Further research is required to elucidate the nature of the differences across scenarios.

From a policy making perspective, the data in Figure 2 communicate two important points. First, support for a tax-funded program of unrestricted access to AD medications is likely to increase as the efficacy of AD medications improves. Second, supporters of the program would generally prefer to pay lower, rather than higher, taxes for the program, regardless of drug efficacy.

Interestingly, at higher taxation amounts (i.e., $225 or greater [Figure 2]), the data suggest a 'floor effect' in each scenario. A subgroup of the population is insensitive to changes in the range of taxation measured in the study. Also, another subgroup exists that would balk at paying ever higher amounts of tax for a program that they support in principle.

Future research would be advisable to set the study findings into a comparative policy context. For example, a group of people who are similar to the participants in this study could be asked whether they would support tax increases for unrestricted access to medications for AD, as well as to medications for other diseases, e.g., cardiovascular disease, diabetes. Policy makers require such comparative data to guide decision making. Another area of future research would be to investigate the determinants of support for specific dollar values of tax increases.

## Conclusion

Four out of five persons in a sample of the adult Canadian general public reported they would support a tax increase to fund unrestricted access to a hypothetical, new AD medication. The highest level of support occurred when the medication was capable of modifying the course of disease without entailing adverse effects. A majority of the sample indicated they would be willing to pay an additional $150 per annum in personal income taxes to fund unrestricted access. However, willingness decreased for amounts over $150.

## Competing interests

The authors declare that they have no competing interests.

## Authors' contributions

MO was involved in the conceptualization, writing, and analysis of the manuscript. JET contributed to conceptualization and analysis, and critically revised drafts of the manuscript. NC cleaned the data, assisted with analysis, and critically revised drafts of the manuscript. PR critically reviewed the manuscript for important scientific content and contributed to the interpretation of the data. All authors read and approved the final manuscript.

## Pre-publication history

The pre-publication history for this paper can be accessed here:

http://www.biomedcentral.com/1472-6963/9/246/prepub

## Supplementary Material

Additional file 1 **Survey**. Copy of the English-language survey of the Canadian general public. Click here for file

## References

[B1] Burns A Iliffe S Alzheimer's disease BMJ 2009 338 467 471 10.1136/bmj.b158 19196745

[B2] Alzheimer's Association 2009 Alzheimer's disease facts and figures Alzheimers Dement 2009 5 234 270 10.1016/j.jalz.2009.03.001 19426951

[B3] Gauthier S Advances in the pharmacotherapy of Alzheimer's disease CMAJ 2002 166 616 623 11898943 PMC99406

[B4] Raina P Santaguida P Ismaila A Patterson C Cowan D Levine M Booker L Oremus M Effectiveness of cholinesterase inhibitors and memantine for treating dementia: Evidence review for a clinical practice guideline Ann Intern Med 2008 148 379 397 18316756 10.7326/0003-4819-148-5-200803040-00009

[B5] Folstein MF Folstein SE McHugh PR "Mini-mental state". A practical method for grading the cognitive state of patients for the clinician J Psychiatr Res 1975 12 189 198 10.1016/0022-3956(75)90026-6 1202204

[B6] Ontario Ministry of Health and Long-term Care Comparative Drug Index - March 3, 2009 Update - Ontario Drug Benefit Program https://www.healthinfo.moh.gov.on.ca/formulary Accessed on: March 18, 2009

[B7] Canadian Study of Health and Aging Canadian Study of Health and Aging: Study methods and prevalence of dementia CMAJ 1994 150 899 913 8131123 PMC1486712

[B8] Zarit S Orr N Zarit J The Hidden Victims of Alzheimer's Disease: Families Under Stress 1985 New York: New York University Press

[B9] Dieckmann L Zarit SH Zarit JM Gatz M The Alzheimer's Disease Knowledge Test Gerontologist 1988 28 402 407 3396921 10.1093/geront/28.3.402

[B10] EuroQol Group EuroQol: a new facility for the measurement of health-related quality of life Health Policy 1990 16 199 208 10.1016/0168-8510(90)90421-9 10109801

[B11] Shaw JW Johnson JA Coons SJ US valuation of the EQ-5D health states: development and testing of the D1 valuation model Med Care 2005 43 203 220 10.1097/00005650-200503000-00003 15725977

[B12] Bonato D Nocera S Telser H The Contingent Valuation Method in Health Care: An Economic Evaluation of Alzheimer's Disease 2001 Bern: Volkswirtschaftliches Institut - Universitat Bern 15027158

[B13] Oremus M Tarride JE A systematic review of the use of contingent valuation in Alzheimer's disease research Dement Int J Soc Res Pract 2008 7 461 480

[B14] Dillman DA Smyth JD Christian LM Internet, Mail and Mixed-Mode Surveys: The Tailored Design Method 2009 3 Hoboken, NJ: John Wiley & Sons, Inc

[B15] Hosmer DW Lemeshow S Applied Logistic Regression 2000 2 Hoboken, NJ: John Wiley and Sons, Inc full_text

[B16] Laurin A International Tax Burdens: Single Individuals with or without Children. PRB-05-107E Ottawa: Parliamentary Information and Research Service http://www.parl.gc.ca/information/library/prbpubs/prb05107-e.pdf Accessed on: September 10, 2006

[B17] Nocera S Bonato D Telser H The contingency of contingent valuation. How much are people willing to pay against Alzheimer's disease? Int J Health Care Finance Econ 2002 2 219 240 10.1023/A:1020441726964 14625942

[B18] Negrin MA Pinilla J Leon CJ Willingness to pay for alternative policies for patients with Alzheimer's Disease Health Econ Policy Law 2008 3 257 275 18634619 10.1017/S1744133108004489

[B19] Werner P Schnaider-Beeri M Aharon J Davidson M Family caregivers' willingness to pay for drugs indicated for the treatment of Alzheimer's disease Dementia 2002 1 59 74 10.1177/147130120200100109

[B20] Statistics Canada Age and Sex Highlight Tables, 2006 Census Ottawa: Statistics Canada http://ceps.statcan.ca/english/census06/data/highlights/agesex/index.cfm?Lang=E Catalogue No. 97-551-XWE2006002, Accessed on: March 24; 2009

