# Supplementary material for: Support for a tax increase to provide unrestricted access to an Alzheimer's disease medication: a survey of the general public in Canada
Source: BMC Health Serv Res. 2009 Dec 29;9:246. doi: 10.1186/1472-6963-9-246 (PMC2806272; doi:10.1186/1472-6963-9-246)
Supplement: Additional file 1 — Survey. Copy of the English-language survey of the Canadian general public. [file 1472-6963-9-246-S1.DOC]

| **GENERAL PUBLIC SURVEY** |
| --- |

**PROGRAMMER: CODE PROVINCE (BASED ON AREA CODE)**

Newfoundland and Labrador 01

Nova Scotia 02

Prince Edward Island 03

New Brunswick 04

Quebec 05

Ontario 06

Manitoba 07

Saskatchewan 08

Alberta 09

British Columbia 10

**INTRODUCTION:**

Hello, my name is [____________________] and I’m calling on behalf of McMaster University. Today we are conducting a research study with Canadians 18 years of age or older about health-related issues in Canada. This research study is funded by the Canadian Institutes of Health Research and has been approved by McMaster University’s Research Ethics Board. Your participation in this study is completely voluntary and your responses will remain strictly confidential. Are you at least 18 years of age?

Yes 1 **CONTINUE**

No 2 **ASK TO SPEAK TO AN ADULT IN THE HOUSEHOLD AND REPEAT INTRODUCTION. SCHEDULE A CALL BACK IF NECESSARY. IF ADULT REFUSES TO PARTICIPATE, THANK AND TERMINATE THE INTERVIEW. RECORD AS REFUSAL IN RECORD OF CONTACTS.**

Great, let’s get started!

A. **[DO NOT ASK]** Record Gender

Male 1

Female 2

1. What is your present age? **PLEASE RECORD EXACT AGE, DO NOT ACCEPT A RANGE**

______ Years

Refused 99 **FOLLOW-UP AND CONFIRM THE RESPONDENT IS 18 YEARS OF AGE OR OLDER. OTHERWISE ASK TO SPEAK WITH AN ADULT IN THE HOUSEHOLD AND REPEAT INTRODUCTION.**

1. What is your current annual household income from all sources, before taxes?

**~IF NECESSARY, TELL RESPONDENTS THAT INCOME IS VERY IMPORTANT TO THE RESEARCHERS DOING THE STUDY BECAUSE THEY ARE LOOKING AT FACTORS THAT PLAY A ROLE IN WHETHER PEOPLE WHO NEED ALZHEIMER’S DISEASE MEDICATIONS CAN ACTUALLY GET THESE MEDICATIONS. MEDICARE DOES NOT PAY FOR THE ENTIRE COST OF THESE MEDICATIONS, SO IT IS IMPORTANT TO UNDERSTAND HOW INCOME MIGHT AFFECT WHETHER PEOPLE CAN GET THESE MEDICATIONS.**

**~IF NECESSARY, REMIND RESPONDENTS THAT THEIR RESPONSES WILL REMAIN STRICTLY CONFIDENTIAL AND ONLY BE REPORTED AT THE GROUP LEVEL, AFTER BEING COMBINED WITH ANSWERS GIVEN BY OTHER RESPONDENTS.**

**~IF ASKED, HOUSEHOLD INCOME FROM ALL SOURCES INCLUDES EARNINGS FROM EMPLOYMENT, INVESTMENTS, REAL ESTATE, INTEREST, PENSIONS, GOVERNMENT SUPPORT PROGRAMS (E.G. EMPLOYMENT INSURANCE, OLD AGE SECURITY), ETC.**

**~READ LIST IF NECESSARY, CODE ONE RESPONSE ONLY**

Less than $20,000 01 **QUOTA = 100**

$20,000 to less than $40,000 02 **QUOTA = 100**

$40,000 to less than $60,000 03 **QUOTA = 100**

$60,000 to less than $80,000 04 **QUOTA = 100**

$80,000 or more 05 **QUOTA = 100**

**[DO NOT READ]** Don’t know/ No answer 98 **THANK AND TERMINATE, RECORD AS DOES NOT QUALIFY IN THE RECORD OF CONTACTS**

**[DO NOT READ]** Refused 99 **FOLLOW UP WITH**, “I understand you do not wish to tell me your household income. I would like to remind you that all your answers will remain strictly confidential. Further, they will only be reported after being combined with other responses. If we are unable to obtain answers to all questions, from all participants, it may affect our ability to form conclusions from the data. Are you still uncomfortable telling me your income?”

Yes 1 **THANK & TERMINATE**

No 2 **RECORD INCOME THEN GO TO Q3**

*Moving along…*

3. For mobility, which of the following statements best describes your own health state today? **READ LIST, CODE ONE RESPONSE ONLY**

I have no problems in walking about 01

I have some problems in walking about 02

I am confined to bed 03

**[DO NOT READ]** Don’t know/ No answer 98

4. In terms of self-care, meaning washing or dressing yourself, which of the following statements best describes your own health state today? **READ LIST, CODE ONLY ONE RESPONSE**

I have no problems with self-care 01

I have some problems washing or dressing myself 02

I am unable to wash or dress myself 03

**[DO NOT READ]** Don’t know/ No answer 98

5. For usual activities such as work, study, housework, family or leisure activities, which of the following statements best describes your own health state today? **READ LIST, CODE ONLY ONE RESPONSE**

I have no problems with performing my usual activities 01

I have some problems with performing my usual activities 02

I am unable to perform my usual activities 03

**[DO NOT READ]** Don’t know/ No answer 98

6. For pain or discomfort, which of the following statements best describes your own health state today? **READ LIST, CODE ONLY ONE RESPONSE**

I have no pain or discomfort 01

I have moderate pain or discomfort 02

I have extreme pain or discomfort 03

**[DO NOT READ]** Don’t know/ No answer 98

7. For anxiety or depression, which of the following statements best describes your own health state today? **READ LIST, CODE ONLY ONE RESPONSE**

I am not anxious or depressed 01

I am moderately anxious or depressed 02

I am extremely anxious or depressed 03

**[DO NOT READ]** Don’t know/ No answer 98

*And next…*

8. To help people say how good or bad a health state is, we would like you to imagine that the best health state possible would score 100 on a scale of 0 to 100. The worst health state possible would score 0. Please indicate on this scale how good or bad your own health state is today, in your opinion. You just have to give a score between 0 and 100.

Score = ______ **RECORD EXACT NUMBER, DO NOT ACCEPT A RANGE**

**[DO NOT READ]** Don’t know/ No answer 998

*Disease Awareness*

(Source: Dieckmann L, Zarit SH, Zarit JM, Gatz M. The Alzheimer’s Disease Knowledge Test. Gerontologist 1988;28:402-407.)

For the next few questions, I am going to read you some statements and I would like you to indicate if you think each statement is true or false.

9. First, Alzheimer’s disease is a normal part of getting older, like gray hair and wrinkles.

True 1 (0 points)

False 2 (1 point)

10. When the husband or wife of an older person dies, the surviving spouse may suffer from a kind of depression that looks like Alzheimer’s disease.

True 1 (0 points)

False 2 (1 point)

11. Stuttering is an inevitable part of Alzheimer’s disease.

True 1 (0 points)

False 2 (1 point)

12. An older man is more likely to develop Alzheimer’s disease than an older woman.

True 1 (0 points)

False 2 (1 point)

13. Nursing home expenses for Alzheimer’s disease patients are covered by the government.

True 1 (0 points)

False 2 (1 point)

Total Score – Questions 9 to 13 (add up number of answers for which 1 point is awarded): ____

*And next…*

14. How would close relatives or friends feel if you supported a tax increase to provide a new medication to persons with Alzheimer’s disease? Would you say they would…**READ LIST, CODE ONLY ONE RESPONSE**

Strongly approve 01

Somewhat approve 02

Somewhat disapprove, or 03

Strongly disapprove 04

**[DO NOT READ]** No opinion 97

**[DO NOT READ]** Don’t know/ No answer 98

*Next I would like to get your opinion on some medications for Alzheimer’s disease. I’m going to read you a few different scenarios and then I’ll ask you to answer a few questions about each.*

**~PLEASE READ EACH OF THE FOLLOWING SCENARIOS TO THE RESPONDENT. READ EACH SCENARIO OR QUESTION AS MANY TIMES AS NECESSARY.**

**~FOR THE ALZHEIMER’S PATIENT IN EACH OF THE SCENARIOS BELOW, USE “HE” IF THE RESPONDENT IS MALE AND “SHE” IF THE RESPONDENT IS FAMALE.**

**Scenario 1:** “Imagine that a new drug for Alzheimer’s disease has come on the market. If a person with Alzheimer’s disease starts taking the drug, then he will have to take one pill every day to benefit. Once on the drug, there is a 65% chance that some of his lost memory will return and some of his behaviour and mood problems will happen less often. Also, he will be able to do some simple tasks that he has not been able to do for awhile (e.g., get dressed). If he does not already experience behaviour and mood problems, or does not have problems doing simple tasks, then he has a 65% chance of avoiding these problems. After about a year, the benefits of the new drug will wear off and his symptoms and problems will come to be like the symptoms and problems of people who do not take the drug.

While he is on the drug, he will not experience any side-effects.

If he does not take the drug, then he will continue to lose memory. In addition, behaviour and mood problems will appear or become worse and he will need more and more help with simple daily tasks.

The government does not currently pay for this new drug. If the government were to fund unrestricted access to the new drug for persons with Alzheimer’s disease, then everyone’s annual taxes would have to increase. Your tax increase would be the equivalent of supplying one person with the drug for one month. If your taxes increase, then you will have less money to spend on other things.”

15. Do you support an overall annual increase in taxes to provide the new medication to persons with Alzheimer’s disease? **IF RESPONDENT SAYS “DEPENDS” RECORD AS YES.**

Yes 1 **CONTINUE**

No 2 **SKIP TO NEXT SCENARIO**

16. Would you support an annual tax increase of $75 to provide the new medication to persons with Alzheimer’s disease? This is equivalent to an extra $6.25 a month.

Yes 1

No 2

17. Would you support an annual tax increase of $150 to provide the new medication to persons with Alzheimer’s disease? This is equivalent to an extra $12.50 a month.

Yes 1

No 2

18. Would you support an annual tax increase of $225 to provide the new medication to persons with Alzheimer’s disease? This is equivalent to an extra $18.75 a month.

Yes 1

No 2

**~IF THE RESPONSES TO Q16, Q17, AND Q18 WERE ALL ‘YES,’ THEN CONTINUE.**

**~IF THE RESPONSES TO Q16, Q17, AND Q18 WERE ALL ‘NO,’ THEN SKIP TO Q20.**

**~IF THE RESPONSES TO Q16, Q17, AND Q18 WERE A MIX OF ‘YES’ AND ‘NO’ ANSWERS, THEN SKIP TO THE NEXT SCENARIO.**

19. What is the maximum annual tax increase that you would support for the new drug? **RECORD EXACT DOLLAR AMOUNT, DO NOT ACCEPT A RANGE**

$ ______ **SKIP TO NEXT SCENARIO**

20. Would you support an annual tax increase of less than $75 for the new drug?

Yes 1 **CONTINUE**

No 2 **WTP=$0, SKIP TO NEXT SCENARIO**

21. How much of an annual tax increase would you support for the new drug? Please specify an amount between $1 and $74. **RECORD EXACT DOLLAR AMOUNT, DO NOT ACCEPT A RANGE**

$ ______

**Scenario 2:** “You just answered the questions assuming someone on the drug would not have side-effects. Now, imagine that the benefits of the drug do not change, but the person taking the drug has a 30% chance of suffering from at least one of a number of side-effects, including nausea, vomiting, diarrhea, or dizziness.”

22. Do you support an overall annual increase in taxes to provide the new medication to persons with Alzheimer’s disease? **IF RESPONDENT SAYS “DEPENDS” RECORD AS YES.**

Yes 1 **CONTINUE**

No 2 **SKIP TO NEXT SCENARIO**

23. Would you support an annual tax increase of $75 to provide the new medication to persons with Alzheimer’s disease? This is equivalent to an extra $6.25 a month.

Yes 1

No 2

24. Would you support an annual tax increase of $150 to provide the new medication to persons with Alzheimer’s disease? This is equivalent to an extra $12.50 a month.

Yes 1

No 2

25. Would you support an annual tax increase of $225 to provide the new medication to persons with Alzheimer’s disease? This is equivalent to an extra $18.75 a month.

Yes 1

No 2

**~IF THE RESPONSES TO Q23, Q24, AND Q25 WERE ALL ‘YES,’ THEN CONTINUE.**

**~IF THE RESPONSES TO Q23, Q24, AND Q25 WERE ALL ‘NO,’ THEN SKIP TO Q27.**

**~IF THE RESPONSES TO Q23, Q24, AND Q25 WERE A MIX OF ‘YES’ AND ‘NO’ ANSWERS, THEN SKIP TO NEXT SCENARIO.**

26. What is the maximum annual tax increase that you would support for the new drug? **RECORD EXACT DOLLAR AMOUNT, DO NOT ACCEPT A RANGE**

$ ______ **SKIP TO NEXT SCENARIO**

27. Would you support an annual tax increase of less than $75 for the new drug?

Yes 1 **CONTINUE**

No 2 **WTP=$0, SKIP TO NEXT SCENARIO**

28. How much of an annual tax increase would you support for the new drug? Please specify an amount between $1 and $74. **RECORD EXACT DOLLAR AMOUNT, DO NOT ACCEPT A RANGE**

$ ______

**Scenario 3:** “Imagine that a new drug for Alzheimer’s disease has come on the market. If a person with Alzheimer’s disease starts taking the drug, then he will have to take one pill every day to benefit. Once on the drug, there is a 65% chance that he will not experience further memory loss. As well, there is a 65% chance that no, or no new, behaviour and mood problems will occur, and that he will continue to perform the simple tasks that he can perform today (e.g., get dressed). After about a year, he will be at the same level of illness as today. This differs from the first two scenarios, where after one year on the drug the person does not get sicker. That is, he will be no sicker today, but no better either.

While he is on the drug, he will not experience any side-effects.

If he does not take the drug, then he will continue to lose memory. In addition, behaviour and mood problems will appear or become worse and he will need more and more help with simple daily tasks.

The government does not currently pay for this new drug. If the government were to fund unrestricted access to the new drug for persons with Alzheimer’s disease, then everyone’s annual taxes would have to increase. Your tax increase would be the equivalent of supplying one person with the drug for one month. If your taxes increase, then you will have less money to spend on other things.”

29. Do you support an overall annual increase in taxes to provide the new medication to persons with Alzheimer’s disease? **IF RESPONDENT SAYS “DEPENDS” RECORD AS YES.**

Yes 1 **CONTINUE**

No 2 **SKIP TO NEXT SCENARIO**

30. Would you support an annual tax increase of $75 to provide the new medication to persons with Alzheimer’s disease? This is equivalent to an extra $6.25 a month.

Yes 1

No 2

31. Would you support an annual tax increase of $150 to provide the new medication to persons with Alzheimer’s disease? This is equivalent to an extra $12.50 a month.

Yes 1

No 2

32. Would you support an annual tax increase of $225 to provide the new medication to persons with Alzheimer’s disease? This is equivalent to an extra $18.75 a month.

Yes 1

No 2

**~IF THE RESPONSES TO Q30, Q 31, AND Q32 WERE ALL ‘YES,’ THEN CONTINUE.**

**~IF THE RESPONSES TO Q30, Q 31, AND Q32 WERE ALL ‘NO,’ THEN SKIP TO Q34.**

**~IF THE RESPONSES TO Q30, Q 31, AND Q32 WERE A MIX OF ‘YES’ AND ‘NO’ ANSWERS, THEN SKIP TO THE NEXT SCENARIO.**

33. What is the maximum annual tax increase that you would support for the new drug? **RECORD EXACT DOLLAR AMOUNT, DO NOT ACCEPT A RANGE**

$ ______ **SKIP TO NEXT SCENARIO**

34. Would you support an annual tax increase of less than $75 for the new drug?

Yes 1 **CONTINUE**

No 2 **WTP=$0, SKIP TO NEXT SCENARIO**

35. How much of an annual tax increase would you support for the new drug? Please specify an amount between $1 and $74. **RECORD EXACT DOLLAR AMOUNT, DO NOT ACCEPT A RANGE**

$ ______

**Scenario 4:** “You just answered the questions assuming someone on the drug would not have side-effects. Now, imagine that the benefits of the drug do not change, but the person taking the drug has a 30% chance of suffering from at least one of a number of side-effects, including nausea, vomiting, diarrhea, or dizziness. Remember that the person taking the drug does not get sicker after one year on the drug, but he does not get any better either.”

36. Do you support an overall annual increase in taxes to provide the new medication to persons with Alzheimer’s disease? **IF RESPONDENT SAYS “DEPENDS” RECORD AS YES.**

Yes 1 **CONTINUE**

No 2 **SKIP TO Q43**

37. Would you support an annual tax increase of $75 to provide the new medication to persons with Alzheimer’s disease? This is equivalent to an extra $6.25 a month.

Yes 1

No 2

38. Would you support an annual tax increase of $150 to provide the new medication to persons with Alzheimer’s disease? This is equivalent to an extra $12.50 a month.

Yes 1

No 2

39. Would you support an annual tax increase of $225 to provide the new medication to persons with Alzheimer’s disease? This is equivalent to an extra $18.75 a month.

Yes 1

No 2

**~IF THE RESPONSES TO Q37, Q38, AND Q39 WERE ALL ‘YES,’ THEN CONTINUE.**

**~IF THE RESPONSES TO Q37, Q38, AND Q39 WERE ALL ‘NO,’ THEN SKIP TO Q41.**

**~IF THE RESPONSES TO Q37, Q38, AND Q39 WERE A MIX OF ‘YES’ AND ‘NO’ ANSWERS, THEN SKIP TO Q43.**

40. What is the maximum annual tax increase that you would support for the new drug? **RECORD EXACT DOLLAR AMOUNT, DO NOT ACCEPT A RANGE**

$ ______ **SKIP TO Q43**

41. Would you support an annual tax increase of less than $75 for the new drug?

Yes 1 **CONTINUE**

No 2 **WTP=$0, SKIP TO Q43**

42. How much of an annual tax increase would you support for the new drug? Please specify an amount between $1 and $74. **RECORD EXACT DOLLAR AMOUNT, DO NOT ACCEPT A RANGE**

$ ______

**43. ASK THIS QUESTION IF RESPONDENT ANSWERED “NO” TO Q15, Q22, Q29, AND Q36. IF RESPONDENT ANSWERED “YES” TO AT LEAST ONE OF THESE FOUR QUESTIONS, THEN SKIP TO Q45**. For each of the four scenarios, you said you would not support an overall annual increase in taxes to provide new medications to persons with Alzheimer’s disease. Could you please explain why? **PROBE AND CLARIFY AS REQUIRED.**

|  |
| --- |

Instructions to Interviewer

**PLEASE CAREFULLY READ THE FOLLOWING DESCRIPTION TO THE RESPONDENT AND ASK ALL 5 QUESTIONS THAT FOLLOW.**

**(Source:** [**http://www.helpguide.org/elder/alzheimers_disease_symptoms_stages.htm**](http://www.helpguide.org/elder/alzheimers_disease_symptoms_stages.htm)**; accessed July 20, 2007.)**

Alzheimer’s disease is a progressive illness that causes memory loss and other cognitive deficits, advancing to major personality changes and eventual loss of control over bodily functions. In the moderate, mid-stage of Alzheimer's disease, mental abilities decline, personality changes, and physical problems develop so that the person becomes more and more dependent on caregivers.

Now, imagine that you have moderate Alzheimer’s disease. How would you answer the following five questions?

44. For mobility, please indicate which statement best describes your own health state today. **READ LIST,** **CODE ONLY ONE RESPONSE**

I have no problems in walking about 01

I have some problems in walking about 02

I am confined to bed 03

**[DO NOT READ]** Don’t know/ No answer 98

45. For self-care, please indicate which statement best describes your own health state today. **READ LIST,** **CODE ONLY ONE RESPONSE**

I have no problems with self-care 01

I have some problems washing or dressing myself 02

I am unable to wash or dress myself 03

**[DO NOT READ]** Don’t know/ No answer 98

46. For usual activities such as work, study, housework, family or leisure activities, which of the following statements best describes your own health state today. **READ LIST,** **CODE ONLY ONE RESPONSE**

I have no problems with performing my usual activities 01

I have some problems with performing my usual activities 02

I am unable to perform my usual activities 03

**[DO NOT READ]** Don’t know/ No answer 98

47. For pain or discomfort, please indicate which statement best describes your own health state today. **READ LIST,** **CODE ONLY ONE RESPONSE**

I have no pain or discomfort 01

I have moderate pain or discomfort 02

I have extreme pain or discomfort 03

**[DO NOT READ]** Don’t know/ No answer 98

48. For anxiety or depression, please indicate which statement best describes your own health state today. **READ LIST,** **CODE ONLY ONE RESPONSE**

I am not anxious or depressed 01

I am moderately anxious or depressed 02

I am extremely anxious or depressed 03

**[DO NOT READ]** Don’t know/ No answer 98

49. Recall the earlier question about rating health states between 0 and 100. The best health state would score 100. The worst health state would score 0. Please indicate on this scale how good or bad your own health state would be today, in your opinion, if you had moderate Alzheimer’s disease. You just have to give a score between 0 and 100.

Score = ______ **RECORD EXACT NUMBER, DO NOT ACCEPT A RANGE**

**[DO NOT READ]** Don’t know/ No answer 998

50. Do you have any family members or close friends that have been diagnosed with Alzheimer’s disease? **IF RESPONDENT INDICATES THAT THE PERSON(S) IS DECEASED, RECORD AS YES.**

Yes (family member) 1

Yes (close friend) 2

No 3

We’re almost finished! I just have a few more demographic questions for statistical purposes. **IF NECESSARY, REMIND RESPONDENTS THAT THEIR RESPONSES WILL REMAIN STRICTLY CONFIDENTIAL AND ONLY REPORTED AT THE GROUP LEVEL, AFTER BEING COMBINED WITH ANSWERS GIVEN BY OTHER RESPONDENTS.**

51. What is the highest level of education that you have completed? **CODE ONLY ONE RESPONSE, READ CATEGORIES IF NECESSARY**

Less than high school 01

Completed high school 02

Some technical/ community college 03

Completed technical/ community college 04

Some university 05

Completed university 06

Some post-graduate studies 07

Completed post-graduate studies 08

**[DO NOT READ]** Don’t know/ No answer 99

52. Are you currently …**READ LIST, CODE ONLY ONE RESPONSE**

Employed full-time (i.e., at least 35 hours/week) 01 **CONTINUE**

Employed part-time (i.e., less than 35 hours/week) 02 **CONTINUE**

**[DO NOT READ]** A student 03 **SKIP TO END**

**[DO NOT READ]** Retired 04 **CONTINUE**

**[DO NOT READ]** Unemployed 05 **SKIP TO END**

**[DO NOT READ]** A homemaker 06 **SKIP TO END**

**[DO NOT READ]** Other (Please specify: __________________) 90 **SKIP TO END**

**END:** That’s all the questions I have for you at this time. Thank you for participating in this important research study.
